# Canakinumab for the treatment of different refractory autoinflammatory disorders

**DOI:** 10.1186/1546-0096-12-S1-P244

**Published:** 2014-09-17

**Authors:** Ingrid HR Grein, Thais C Meneghetti, Christina F Pelajo, Loris L Janz Junior, Marcia Bandeira

**Affiliations:** 1 Reumatopediatria, Hospital Pequeno Príncipe, Curitiba, Brazil

## Introduction

Interleukin-1β (IL-1β) is a powerful pro-inflammatory cytokine synthesized in the inflammasome complex of immune cells in response to injury and infection. The excess activity of this cytokine plays a fundamental role in the pathogenesis of the autoinflammatory disorders. An emphasis on pediatric rheumatology is given to systemic-onset juvenile idiopathic arthritis (SoJIA) and periodic fever syndromes. Until the last decade, there wasnt an efficient alternative for the treatment of autoinflammatory syndromes refractory for convencional therapies. This scenery was modified with the introduction of biological agents, especially the IL-1 antagonist group (Anakinra, Rilonacept and Canakinumab). Canakinumab, a fully human anti-IL-1β monoclonal antibody, selectively binds to IL-1β, and intercepts the spread of inflammation. This medication was approved for treatment of cryopyrin-associated periodic syndrome (CAPS) in 2009, and SoJIA in 2013, and has shown to be an excellent tool for controlling these disorders.

## Objectives

To report the clinical and laboratorial response to the use of Canakinumab in pediatric patients with distinct autoinflammatory syndromes.

## Methods

We performed a retrospective chart review of patients in use of Canakinumab in monitoring at the Rheumatology Department of a tertiary center in Curitiba (Brazil). Patients with arthritis (SoJIA and pediatric granulomatous arthritis) received a subcutaneous dose of 4mg/kg every 4 weeks, whereas patients with periodic fever syndromes received a subcutaneous dose of 2mg/kg every 8 weeks. The clinical and laboratorial activity of these diseases were evaluated right after the first medication dose. The patients were monitored during the next years in order to evaluate the long-term response to Canakinumab.

## Results

We reported 5 cases of autoinflammatory disorders: 2 cases of SoJIA, 1 neonatal-onset multisystem inflammatory disorder (NOMID), also known as chronic infantile neurological cutaneous and articular syndrome (CINCA), 1 familial Mediterranean fever (FMF) and 1 pediatric granulomatous arthritis (Blau Syndrome). All cases showed an excelent clinical and laboratorial response after Canakinumab first dose. One SoJIA patient reported flare during the NSAID and corticoid tapering. With the reintroduction of NSAID, the patient satisfactory responded to the medication and the disease became inactive again. The Blau Syndrome patient had a flare after withdrawal therapy for 5 months, due to failure to receive the medication. As soon as Canakinumab was reintroduced, the disease was also remissioned. Neither patients showed adverse effects.

## Conclusion

Although randomized, double-blind, controlled studies are still needed, the anti-IL-1β antibody Canakinumab has proven to be an excellent alternative for refractory autoinflammatory disorders, due to its high efficacy and safety.

## Disclosure of interest

None declared.

